# Identifying relevant medical reports from an assorted report collection using the multinomial naïve Bayes classifier and the UMLS

**Published:** 2007

**Authors:** Vijayaraghavan Bashyam, Craig Morioka, Suzie El-Saden, Alex AT Bui, Ricky K Taira

**Affiliations:** 1 Department of Information Studies, University of California - Los Angeles, Los Angeles, CA 90024; 2 Department of Radiological Sciences, University of California - Los Angeles, Los Angeles, CA 90024

**Keywords:** Document Classification, Information Filtering, Content Analysis, Naïve Bayes, Medical Reports

## Abstract

A patient’s electronic medical record contains a large number of medical reports and imaging studies. Identifying the relevant information in order to make a diagnosis can be a time consuming process that can easily overwhelm the physician. Summarizing key clinical information for physicians evaluating brain tumor patients is an ongoing research project at our institution. Notably, identifying documents associated with brain tumor is an important step in collecting the data relevant for summarization. Current electronic medical record systems lack meta-information which is useful in structuring heterogeneous medical information. Thus, identifying reports relevant to a particular task cannot be easily retrieved from a structured database. This necessitates content analysis methods for identifying relevant reports. This paper reports a system designed to identify brain-tumor related reports from an assorted collection of clinical reports. A large collection of clinical reports was obtained from our university hospital database. A domain expert manually annotated the documents classifying them into `related’ and ùnrelated’ categories. A multinomial naïve Bayes classifier was trained to use word level and UMLS concept level features from the reports to identify brain tumor related reports from the assorted collection. The system was trained on 90% and tested on 10% of the manually annotated corpus. A ten-fold cross validation is reported. Performance of the system was best (f-score 94.7) when the system was trained using both word level and UMLS concept level features. Using UMLS concepts improved classifier accuracy.

## Introduction

Electronic medical records (EMRs) are increasingly being adopted by healthcare centers in the United States [1]. A typical EMR consists of both unstructured and structured data. Structured data usually comprises patient demographics such as name, age, patient-id, sex, social security number, etc. Unstructured data consists of free-text medical reports and images [2]. Each visit to the hospital can generate several medical reports depending on the extent of the healthcare provided to the patient. Thus, an elderly or a chronic diseased patient is likely to have several hundred medical reports accumulated over many hospitals visits [3]. While records of the same patient are not typically shared electronically among healthcare centers (nor centrally located in one place) [4], it is common for patients to have an extensive medical history at a particular hospital that they regularly visit for treatment.

Analyzing a patient’s history is an important aspect of the diagnosis, prognosis and patient evaluation process [5–7]. Hence, the physician often has to peruse through a large volume of textual reports while diagnosing a patient. This process is cumbersome and time consuming. Summarizing the patient information can be very useful since physicians rarely have the time to manually inspect the entire patient record. In previous work, we have built a system that creates a problem-centric summarization of the electronic medical record. Identifying relevant medical reports for summarization is a key preprocessing step in this process. Unfortunately, EMR systems are still not at the level of advancement where structured (meta) information regarding the content of a report is systematically stored in a database [8]. Therefore analyzing report content is the often resorted method for identifying relevant medical documents or patients fitting particular medical criteria [9]. In this paper we describe a system that uses document classification methods to identify *brain tumor*-related medical reports from an assorted set of reports. This system is intended to be a preprocessing filtering module to our document summarization and visualization system. The remainder of this paper is organized as follows: Section 2 reviews the relevant literature in biomedical text classification and introduces the multinomial naïve Bayes classifier; Section 3 describes the data collection, classifier training and classifier testing methods; Section 4 reports the results and discusses the results and ends with an overview of the future proposed goals for this project.

## Background

The Background section is organized as follows. We first review the relevant text classification literature focusing on applications in biomedical text analysis. This subsection is followed by an introduction to the multinomial naïve Bayes classifier which is the optimal classifier for our problem.

### Text Classification:

Text classification has been an area of research since 1960, reaching a point where it became a major subfield in the 1990s [10]. Several machine learning methods have been applied for both binary and multi-category text classification with features ranging from: words [11]; words with frequency derived weighting [12]; natural language processing (NLP) derived features such as noun phrases [13]; and features derived using domain knowledge[14]. The appropriateness of the classifier used depends largely on the problem and feature distribution in the dataset. In general, incorporating domain knowledge has been shown to improve the performance of machine learning algorithms [15].

In the biomedical domain, there have been some efforts in applying machine learning methods for identifying relevant documents. Wilcox et al. [16] used domain knowledge and NLP derived features to identify six clinical conditions in chest radiograph reports. They report significant improvement in classifier performance using domain knowledge for multiple classifiers such as MC4 decision tree, CN2 induction, naïve Bayes, IB nearest neighbor, and the decision tables algorithm. Herron [17] reported the use of WordNet and the Unified Medical Language System (UMLS) for feature representation for automatic classification of consumer health web sites into topic categories. He used support vector machines for multiple binary classification tasks. The performance of the system largely depended on the target class. The weakest result came from the most frequent topic category [17]. However, he noted that use of WordNet relations improved performance. Chapman et al. [18] used three methods – rule-based, decision trees, and Bayesian networks to automatically identify chest radiography reports supporting acute bacterial pneumonia. NLP-coded domain specific concepts were used as features. High performance (72%−92% precision/recall depending on the method) was reported for all the three systems.

### Classifier Review:

The naïve Bayes classifier is probably the most frequently used classifier in machine learning [19], which is based on the simple well-known Bayes Theorem of Probability [20]. The multinomial naïve Bayes approach to text classification uses vectors of term counts to represent documents. It is assumed that each class can be described as a multinomial distribution. The naïve Bayes assumption is that each word in the document is generated independent from each other, i.e., the probability of the occurrence of each term event in a document is independent of the context and position in the document. The frequency count of the word in a class is used to determine the probability of a document belonging to a particular class. To illustrate, let *p*(*w_i_* ∣ *C*) represents the probability that the i^th^ word (*w*_*i*_) of a document occurs from class *C*. Let *f*_*i*_ denote the frequency count of word *w*_*i*_ in that class. Then the likelihood of a document given a class C can be written as:

(1) $$p(D|C)=\frac{\left(\sum_{i}f_{i}\right)!}{\prod_{i}f_{i}!}\prod_{i}{\left(p\left(w_{i}|C\right)\right)}^{f_{i}}$$

By assigning a prior distribution over the set of classes *p(C)*, we arrive at the least-error classification rule which selects the class with the largest posterior probability. Assuming that there are two classes A and B the final classification rule is given by:

(3) $$\begin{array}{cc} Category\;A & \begin{array}{l} \;\text{if}\;\text{ln}\frac{p(D|A)}{p(D|B)}+\text{ln}\frac{p(A)}{p(B)}>0 \end{array}\\ Category\;B & otherwise \end{array}$$

Multinomial Bayesian models have been used for several classification tasks including hierarchical organization of text databases [21], word-sense disambiguation [22] and document categorization [23–25],

## Methods

### Data Collection:

**Figure 1:**
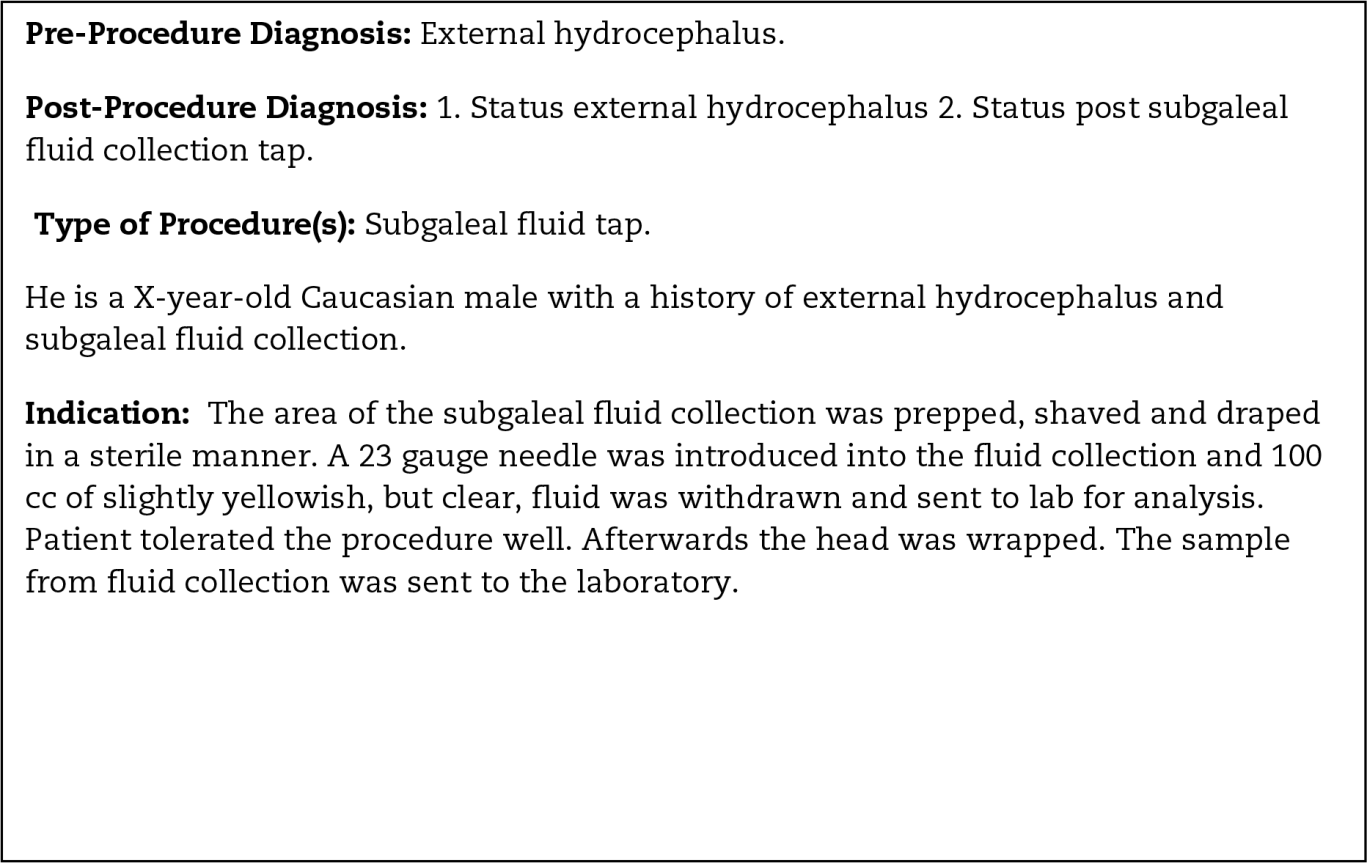
Sample text from a brain tumor related surgery report

**Figure 2:**
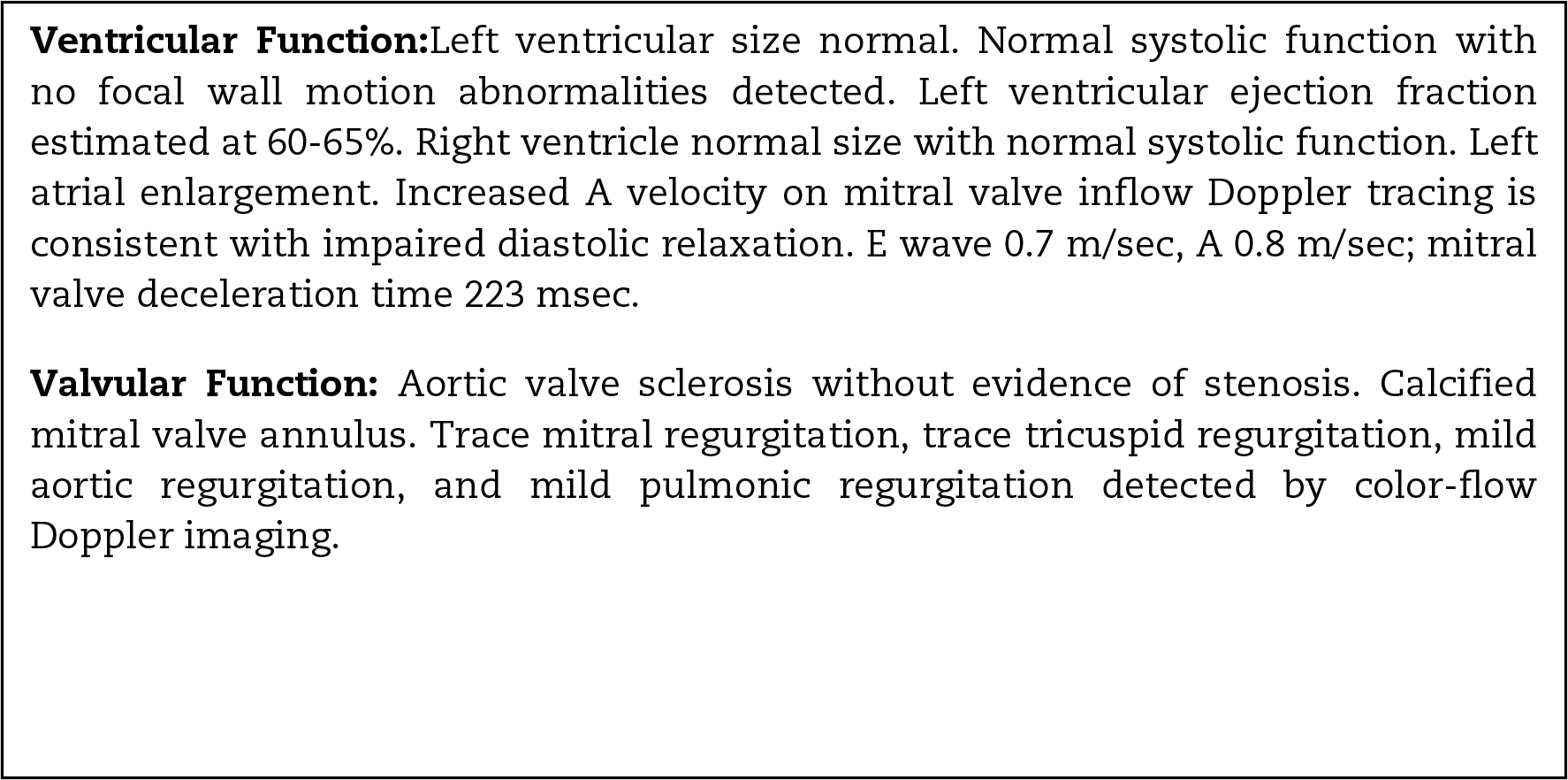
Sample text from a cardiography report unrelated to brain-tumor

We used the open source XML gateway DataServer to interface with hospital databases [26]. DataServer is middleware, situated between clients and traditional health information systems (HIS), radiology information systems (RIS) and picture archive and communication systems (PACS). A suite of web-based tools allows for the centralized management of distributed data sources. DataServer is used for aggregating XML-based patient medical records, in both clinical and research applications at our institution. Using this interface, a large corpus of 1197 documents was extracted from our university-hospital’s database. The documents included several kinds of medical reports - radiology, pathology, discharge summaries, surgery, electrocardiography and radiation oncology reports. A physician manually inspected this corpus, identifying 805 documents pertaining to brain tumor and 392 documents unrelated to brain tumor. Sample text from reports related and unrelated to brain tumor can be seen in Figures 1 and 2, respectively. Report contents have been deidentified to protect patient privacy.

### Feature Selection:

In any pattern classification task, it is important to compile a large number of quality training examples that reflect the underlying distribution of the pool statistics. A representative sample of training data is important as the training examples reflect exactly how the classifier will behave. Thus, feature selection is an important part of the training process. In the world of text categorization, words are the most commonly used features. However medical concepts often span multiple words and it is important to identify the right set of terms for classification. For example, the term *heart attack* is very specific to the domain of cardiology whereas the individual words *heart* and *attack* are not as specific. There can be references to the word *heart* in several reports unrelated to cardiology (e.g. the heart is almost always seen in a chest X-Ray and is frequently mentioned in a thoracic radiography report). Therefore using medical terms rather than words, as features may be more appropriate than individual words for content analysis in the clinical domain.

In order to identify clinical concepts, we used a subset of the Unified Medical Language System 2006AB (UMLS) [27] as a lexicon of features. The UMLS is a controlled repository of various biomedical terminological systems maintained by the National Library of Medicine. The UMLS contains over 1.3 million unique concepts with over 6.4 millionunique terms (string entries) from 119 vocabularies in 17 languages. To reduce complexity, we created a subset of UMLS by including only English terms corresponding to semantic types relevant to clinical medicine. A list of the semantic types we used, with example terms is given in Table 1. Overall, our lexicon of clinical terms contained about 700,000 string entries. Identifying the UMLS concepts within a medical report was performed using a left-to-right marching parser based on a modified version of the Aho-Corasick algorithm [28]. The algorithm works by looking up a previously created index for all words beginning with a current word and identifying the longest substring from the returned set. This method is very efficient in identifying longest substrings from a sentence given a lexicon of substrings. A detailed evaluation of an implementation of this system can be found in Bashyam et al. [29].

It is important to note that the features were at the concept level rather than at the term level. By using concept level features, we could ensure that all instances of the synonyms *heart attack, myocardial infarction, AMI, heart infarction,* etc. were represented by the same feature, a single UMLS concept unique identifier (CUI) C0027051, rather than by different strings. Using the CUIs increases the discriminatory power of an individual feature and also reduces the complexity of the classification task by ensuring a smaller feature set.

### Testing:

The Weka implementation [30] of the multinomial naïve Bayes classifier was used for training and testing the manually labeled corpus. In order to ensure that the conclusions drawn from the results of the classification experiment are not *suggested by the data*, a ten-fold cross validation was conducted. The data was randomly partitioned into 10 equal subsets. During each instance, 9 sets were used for training the classifier and the remaining set was used for testing. The average of the 10 results is reported as the final result. A ten-fold cross validation is the commonly used method to evaluate classification results [31].

For comparison purposes, the same corpus was classified using: 1) the traditional word level feature set; 2) the UMLS concept level feature set; and 3) a hybrid feature set incorporating UMLS concepts and words. Stop words were not considered as features.

The performance of the classifier is quantified by the following three metrics: 1) precision, (the percentage of identified brain tumor-related reports that were accurate); 2) recall, (the percentage of brain tumor-related reports in the corpus identified by the classifier); and 3) balanced - f score, (the equally weighted harmonic mean of precision and recall). These are related to the True Positives (TP), False Positives (FP) and False Negatives (FN) as follows:

(4) $$Precision\;=\frac{TP}{TP\;+\;FP}$$

(5) $$Recall\;=\frac{TP}{TP\;+\;FN}$$

(6) $$f_{2}=2\times \frac{Precision\;\times \;Recall}{Precision\;+\;Recall}$$

## Results and Discussion

The performance of the classifier was best (Precision 0.972 / Recall 0.906 / f-score 0.947) when the combination of UMLS concepts and words were used as features. Intuitively this can be interpreted as follows: the classifier is able to correctly identify 90.6% of the existing brain tumor reports from the collection, and 97.2% of all reports identified as brain tumor reports are actually brain tumor reports. The f-score gives equal importance to both precision and recall and is used as a single score to rank the performance. The next best performance came from the classifier trained on word level features (f-score 0.938). Interestingly, the classifier performed relatively poorly achieving an f-score of only 0.903 with purely UMLS CUIs. Tables 2,3 and 4 presents the three confusion matrices and Table 5 summarizes the results. We conducted an error-analysis to investigate why the performance was worse when using ULMS CUIs as the results appears counter-intuitive. The UMLS CUIs represent the bulk of the domain specific information, which actually differentiates the various documents. Thus the best performance can be logically expected when the UMLS CUIs are used as features.

We observed that the medical reports varied in size significantly with the smallest report containing only 32 words (<1kB) and the largest report containing 1551 words (16kB). This variation is due to the fact that the report length depends on the document type. To illustrate, surgery reports are often lengthy with narrative descriptions of the entire surgical process whereas pathology reports are very brief with bulleted points. In addition, different physicians dictate the reports in their own unique ways. Some physicians carefully document their findings whereas some physicians dictate brief overviews. In our entire collection, there were 106 reports that contained no UMLS CUIs due to the brevity of description. Therefore these documents had no features associated with them. This is most likely due to two reasons: 1) the UMLS may not have sufficient coverage of brain tumor-related terminology; and 2) errors in the document generation process (typing, transcription etc) resulting in unknown string forms. Such errors are commonly found in clinical reports [32–34]. We removed the 106 reports and reran the system with the word-level features and the hybrid level features in order to compare with the CUI only set. Upon removing these reports, the performance (f-score) at the word level was 0.94 and at the hybrid level was 0.95 whereas the CUI only level had an f-score of 0.903.

Overall there were 25,804 features at the word-level, 2956 features at the UMLS concept level and 14,847 features at the hybrid level. The hybrid level provides a good balance by incorporating the best features from both approaches. While the UMLS concepts help in reducing the number of features, they are not always found in every report. To make up for this disadvantage, the hybrid approach includes word level features where necessary and hence is able to perform the best.Due to the large differences in the nature of corpora and actual clinical data which exhibit high variability depending on the institutional practices, it is difficult to compare our work with other work in this field. The United States laws relating to patient privacy (HIPAA regulations) [35] make it difficult to create standardized clinical datasets open to the public so that different methods can be tested for comparison.

### Future Work

Our current system is trained to identify only brain-tumor related medical reports. For future work, we propose to categorize documents under multiple categories such as brain diseases (stroke / epilepsy / Alzheimer’s disease), medical specialty (radiology / pathology / surgery) and information novelty (new findings discovered / old findings repeated). This approach would enable the system to identify relevant reports depending on the physician’s need. This would require training the classifier to identify the new classes and testing it on unseen data. In order to improve accuracy, increased training is one possible option. The Naïve Bayes classifier shows much promise for multi-category classification as well [36].

## Conclusion

Text categorization has many potential data acquisition applications in clinical practice as well as in biomedical research. We developed a fast, accurate document filtering system to identify brain tumor related medical reports within a collection of assorted medical reports. High performance accuracy is achieved by a rich set of discriminating domain-specific features and the multinomial naïve Bayes classifier. Using the UMLS CUIs as features along with words helped in significantly reducing the feature space while increasing system performance. The system is proposed to be used as part of a patient information summarization and visualization system for clinical practice.

## Figures and Tables

**Table 1.** Examples of concept level features used in the classification

UMLS Semantic Type	Example

Anatomical Structure	body
Embryonic Structure	arterial canal
Congenital Abnormality	absence of aorta
Acquired Abnormality	skin lesion
Fully Formed Anatomical Structure	male body
Body System	reproductive system
Body Part, Organ, or Organ Component	brain
Body Location or Region	premotor area
Body Space or Junction	thoracic cavity
Finding	biliuria
Injury or Poisoning	mosquito bites
Pathologic Function	pelvic hematoma
Disease or Syndrome	color blindness
Mental or Behavioral Dysfunction	schizophrenia
Cell or Molecular Dysfunction	genetic inversion
Experimental Model of Disease	alloxan diabetes
Sign or Symptom	wrist pain
Anatomical Abnormality	pulmonary valve stenosis
Neoplastic Process	carcinoma

**Table 2.** Confusion Matrix (Word level features)

Classified As →	A	B

A	730	75
B	21	371

A – Brain Tumor Related, B – Unrelated to Brain Tumor

**Table 3.** Confusion Matrix (UMLS CUI features)

Classified As →	A	B

A	645	54
B	85	307

A – Brain Tumor Related, B – Unrelated to Brain Tumor

**Table 4.** Confusion Matrix (Words + UMLS CUI features)

Classified As →	A	B

A	753	52
B	32	360

A – Brain Tumor Related, B – Unrelated to Brain Tumor

**Table 5.** Classifier performance for identifying brain tumor related reports

Features Used	TP	FP	FN	TN	P	R	F

Words	730	21	75	371	0.972	0.9068	0.938
UMLS CUIs	645	85	54	307	0.884	0.9227	0.903
CUIs + Words	753	32	52	360	0.959	0.9354	0.947

TP – True Positives, FP – False Positives, FN – False Negatives, TN – True Negatives

P – Precision, R – Recall, F – Balanced F Score
